# Large‐Peptide Permeation Through a Membrane Channel: Understanding Protamine Translocation Through CymA from *Klebsiella Oxytoca* **

**DOI:** 10.1002/anie.202016943

**Published:** 2021-03-03

**Authors:** Sushil Pangeni, Jigneshkumar Dahyabhai Prajapati, Jayesh Bafna, Mohamed Nilam, Werner M. Nau, Ulrich Kleinekathöfer, Mathias Winterhalter

**Affiliations:** ^1^ Department of Life Sciences and Chemistry Jacobs University 28759 Bremen Germany; ^2^ Department of Physics and Earth Sciences Jacobs University Bremen 28759 Bremen Germany

**Keywords:** electrophysiology, membrane translocation assay, molecular dynamics simulations, outer membrane porins, protamine

## Abstract

Quantifying the passage of the large peptide protamine (Ptm) across CymA, a passive channel for cyclodextrin uptake, is in the focus of this study. Using a reporter‐pair‐based fluorescence membrane assay we detected the entry of Ptm into liposomes containing CymA. The kinetics of the Ptm entry was independent of its concentration suggesting that the permeation through CymA is the rate‐limiting factor. Furthermore, we reconstituted single CymA channels into planar lipid bilayers and recorded the ion current fluctuations in the presence of Ptm. To this end, we were able to resolve the voltage‐dependent entry of single Ptm peptide molecules into the channel. Extrapolation to zero voltage revealed about 1–2 events per second and long dwell times, in agreement with the liposome study. Applied‐field and steered molecular dynamics simulations added an atomistic view of the permeation events. It can be concluded that a concentration gradient of 1 μm Ptm leads to a translocation rate of about one molecule per second and per channel.

## Graphical abstract

Surprisingly, large peptides (Protamine, 5.1 kDa) can permeate through bacterial outer membrane channels. The use of fluorescence, electrophysiology, and all‐atom modeling allows to quantify the flux. This approach can be transferred to related problems.

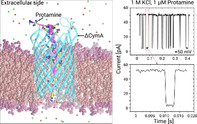

## Introduction

**Figure 1:**
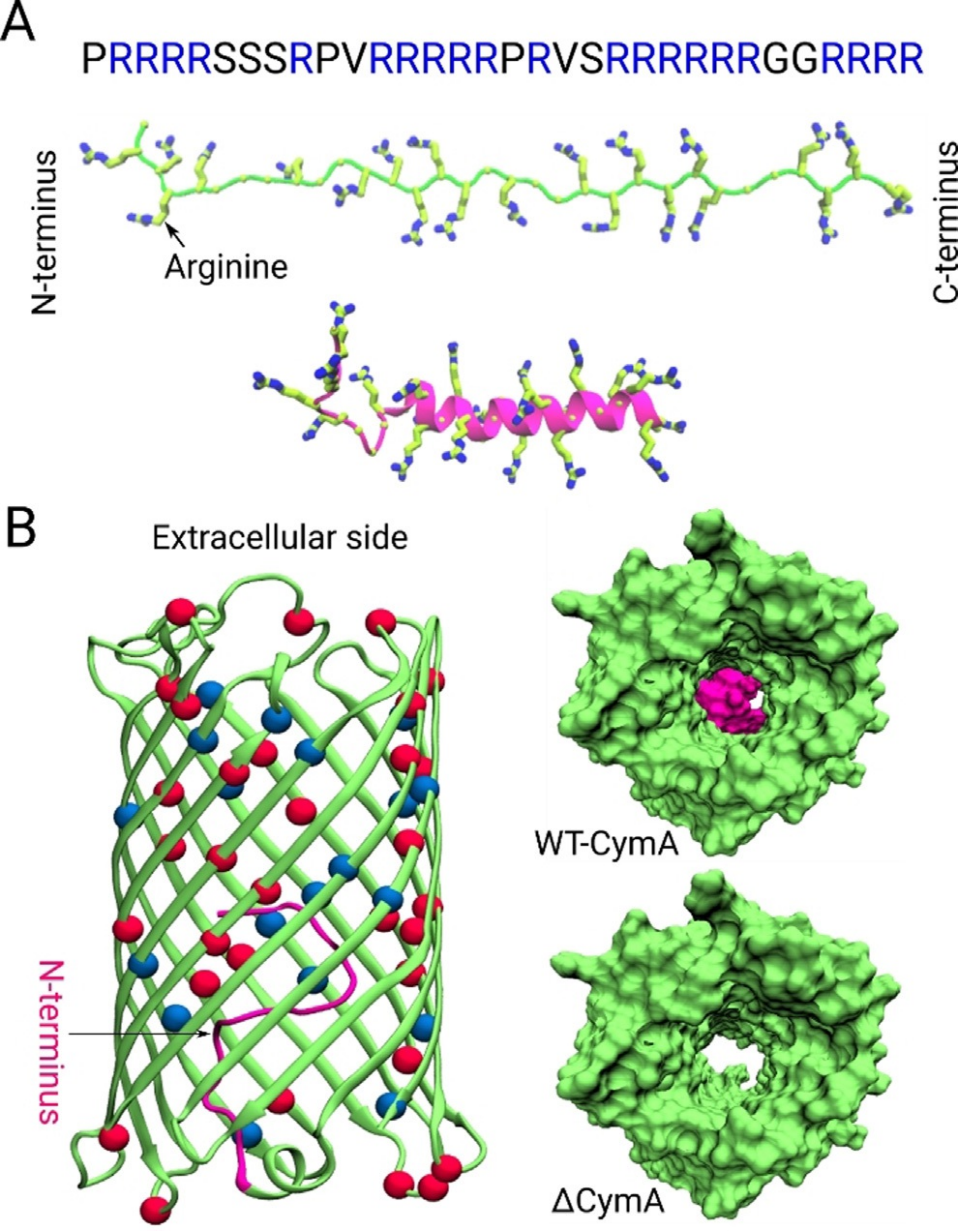
A) Primary amino acid sequence of Ptm (top) and molecular models of its secondary structure in the disordered (middle) and helical conformation (bottom); the arginine residues are highlighted as sticks in both secondary structures. B) Crystal structure of the wild‐type CymA channel from *Klebsiella oxytoca*, shown in cartoon representation in the left part (PDB ID: 4D51). The first 15 residues of the N‐terminus domain that are deleted in the ΔCymA mutant are highlighted in magenta. The 32 acidic residues (ASP, GLU) and the 19 basic residues (ARG, LYS) located at the inner pore surface are highlighted as red and blue beads, respectively. The right panel depicts the interior of the wild‐type (top, CymA) and mutant (bottom, ΔCymA) channels viewed from the extracellular side.

Rapid and label‐free monitoring of drug uptake into Gram‐negative bacteria is a bottleneck in antibiotic drug discovery. The outer membrane of Gram‐negative bacteria acts as a selective barrier for the uptake of small molecules including antibiotics and antimicrobial peptides. The outer membrane itself is nearly impermeable but rich in water‐filled transmembrane proteins called porins that facilitate the passive permeation of small hydrophilic molecules below molecular weights of 600 Da.[^1^, ^2^] In contrast, large molecules are generally excluded but selected ones find their way via self‐promoted uptake.[^2^, ^3^, ^4^, ^5^] Antimicrobial peptides often act via their lytic activity.[^6^, ^7^] Typical examples are cationic antimicrobial peptides that have attracted wider interest as substitutes for classical antibiotics.[^8^, ^9^, ^10^, ^11^, ^12^, ^13^, ^14^, ^15^, ^16^] Whether these polycationic peptides permeate using a self‐promoted pathway or through channel proteins remains an open but crucial question in understanding their antimicrobial activity and other putative functions. One such molecule is protamine (Ptm) which is a 32 amino‐acid‐long polycationic peptide with a molecular mass of 5.1 kDa that contains 21 arginine residues (Figure 1 A). This molecule is a cheap byproduct from fish industry and used as an antibiotic in fish farming.^[17]^ Ptm does not show lytic activity but seems to enter the periplasmic space of *Escherichia coli*, *Salmonella typhimurium*, and *Pseudomonas aeruginosa*.^[5]^ So far, the mode of action, i.e., self‐promoted uptake or uptake via a protein channel, is unknown.

Recently, we qualitatively characterised the permeation of Ptm through OmpF by using a fluorescence assay[^18^, ^19^] which is based on the encapsulation of a host–guest complex between calixarene (CX4) and lucigenin (LCG) into the aqueous interior of vesicles.^[20]^ The host–guest pair has to be selected for negligible membrane permeability and has to be sufficiently large in order not to permeate through bacterial membrane proteins like OmpF.^[19]^ Analytes that permeate across the membrane and bind to the synthetic CX4 receptor (such as cationic peptides) affect a displacement of the dye LCG.[^18^, ^21^] This technique is known as “tandem assay” and has been extensively applied to assess enzyme activity,^[22]^ chirality recognition,^[23]^ and drug delivery,^[24]^ as well as for chemosensing in cells.^[25]^

Herein, we unravel the molecular details of the passive porin uptake of large substrates employing a joint experimental and computational approach. As model porin, we selected CymA from *Klebsiella oxytoca* (Figure 1 B), a channel which allows the passage of large cyclic α‐cyclodextrin molecules (approx. 1 kDa)^[26]^ and which, after deletion of 15 N‐terminal residues, constitutes a cation‐selective hollow channel with a 15 Å diameter termed ΔCymA.[^27^, ^28^] This channel has been considered because the large pore size allowing passive permeation of large cyclic molecules also provides a potential route for the uptake of antimicrobial peptides like Ptm.

First, we used the tandem membrane assay to characterise the Ptm permeation into the interior of the liposome by fluorescence ensemble measurements.^[18]^ To obtain an accurate single‐molecule picture of the translocation, we further employed single‐channel electrophysiology in order to monitor potential translocations across ΔCymA. Finally, and to gain detailed atomistic insight into the translocation mechanism that is elusive to experiments, applied‐field and steered all‐atom MD simulations were performed.

## Results

### Ensemble Measurements of Protamine Translocation through CymA by the Tandem Membrane Assay

**Figure 2:**
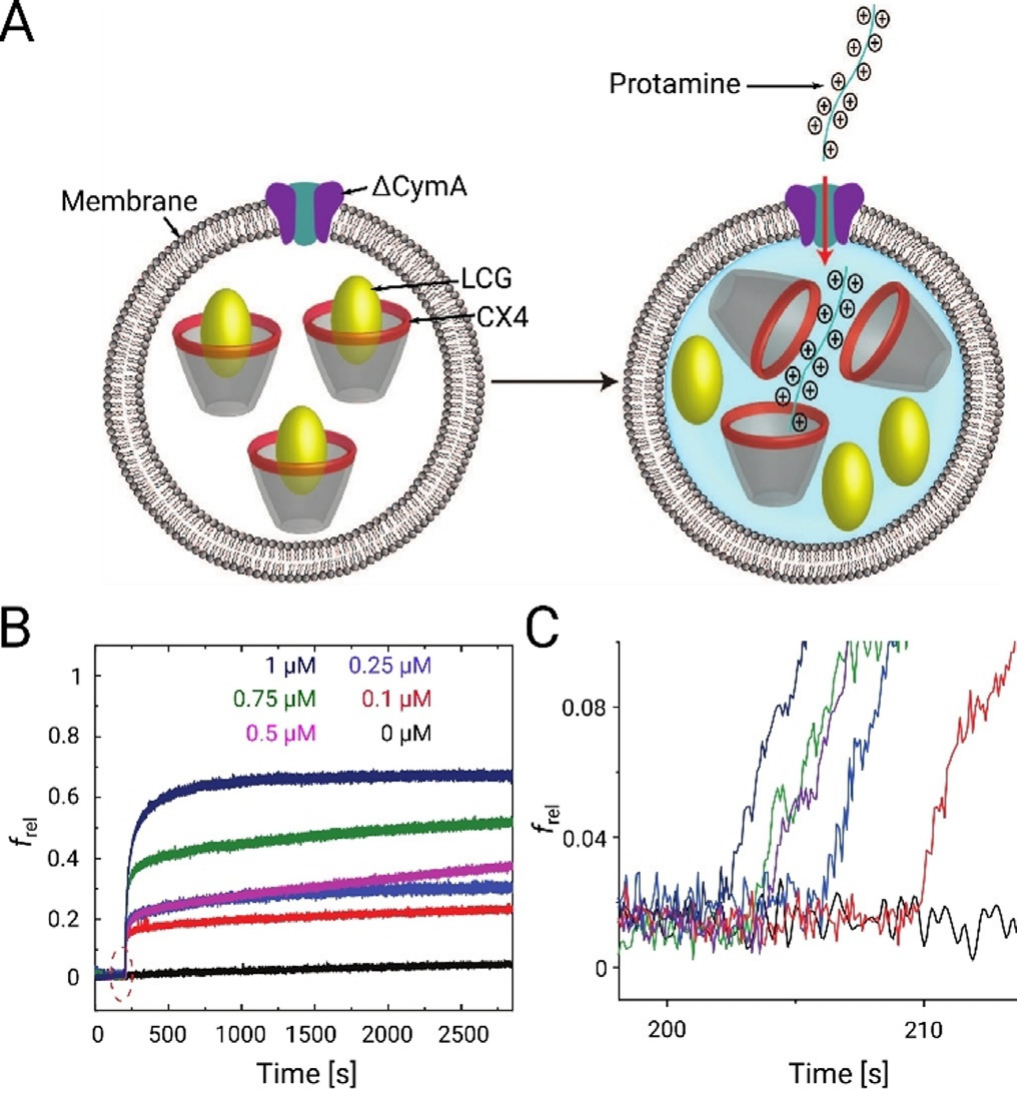
Tandem membrane assay for Ptm translocation across CymA channels. A) Schematic representation illustrating a liposome with a ΔCymA channel embedded into the membrane together with encapsulated fluorophores (LCG) which are being quenched in the presence of complexing calixarenes (CX4). An enlarged concentration of Ptm molecules outside the liposomes can lead to permeation of these molecules into the liposome followed by a fluorophore displacement and a fluorescence increase (indicated by the blue background). B, C) Fluorescence kinetics of Ptm translocations through ΔCymA. B) Time‐dependent *f*_rel_ changes of POPC⊃CX4/LCG liposomes (700 μm CX4, 500 μm LCG, the symbol ⊃ stands for “with encapsulated”) upon addition of 45 nm ΔCymA and different concentrations of Ptm in 10 mm sodium phosphate, pH 7.0. The fluorescence of LCG was monitored at 500 nm, with excitation at 367 nm. C) The initial reaction rate zoomed in from panel (B). The start of the lag phase at different times between 200 and 210 seconds simply shows the (arbitrary) time at which protamine was added manually.

In a first series of experiments, we investigated the translocation of Ptm across the ΔCymA channel by using the previously introduced tandem membrane assay.^[19]^ Briefly, the procedure includes the encapsulation of the CX4/LCG reporter pair into the interior of liposomes as schematically shown in Figure 2 A. Subsequent insertion of ΔCymA channels into the liposome membranes followed by an addition of Ptm is expected to lead to a fluorescence turn‐on response by displacement of the dye by the protamine from the macrocyclic cation receptor CX4 (see Section 1 of the SI for further details).

Fluorescence measurements in two control experiments performed without using Ptm molecules or ΔCymA channels resulted in a flat line excluding a leakage of CX4 or LCG through the pore or a passage of Ptm molecules through the lipid membrane (see Figure S2). Subsequently, during the actual translocation experiments, addition of Ptm molecules and ΔCymA pores in any order led to an initially rapid increase in the fluorescence followed by a slower rise. These results strongly suggest that Ptm permeation occurs selectively through the ΔCymA channels. Subsequently, we performed a set of experiments by varying the Ptm concentration (0.1–1.0 μm) and monitoring the initial rates for the fluorescence increase, which were found to be closely comparable (Figure 2 B with a zoom in Figure 2 C). Accordingly, we suggest that the rate‐limiting step for Ptm passing through the ΔCymA channel is the exit from the channel, most probably due to strong electrostatic interactions of the arginine‐rich Ptm with the acidic residues of the pore (Figure 1 B). From these experiments, the flux for a 1 μm Ptm concentration gradient is estimated to be roughly equivalent to 1–3 Ptm molecules per second through one ΔCymA channel (see Section 1 in the SI for more details). From a biological perspective, the transport through wild‐type (WT) CymA channels has a larger significance. The N‐terminal loop is weakly bound inside the CymA channel and modulates the translocation of large peptides through the porin (Figure 1 B).^[28]^ Indeed, the experiments performed using WT CymA as a pore show also an increase in fluorescence, confirming an influx of Ptm molecules (see Figure S3 in the SI), but the kinetics, as judged from the initial rate, is slightly slower than for the mutant ΔCymA pore lacking the back‐folded loop. Nevertheless, it should be noted that inside a bacterial outer membrane the behavior of the N‐terminal loop might be different or it may have an unknown biological function.

### Determining the Translocation Rates Using Electrophysiology

Single‐channel electrophysiological measurements were performed next in order to investigate the translocation of Ptm molecules through CymA channels at a single molecule level. In essence, a transmembrane electric field is applied to pull the cationic peptide through a single channel reconstituted into a flat lipid bilayer membrane. The entry of each Ptm peptide, which can be resolved at the single‐molecule level with this technique, is observed by a complete ion current blockage. A statistical analysis of the blockage lengths or dwell times as a function of external voltage is used to reveal potential Ptm translocations as well as its kinetics through the CymA channel or its mutant. Concentration‐driven translocation can further be investigated by measuring the reversal potential created by a Ptm sulfate gradient.

Starting with two compartments filled with 1 m KCl, a single WT CymA channel was reconstituted into the DPhPC bilayer from the side that is electrically grounded, also called the *cis* side, and ion current traces were recorded by applying a transmembrane potential, *V*_m_, at the *trans* side (see Section 2 in the SI for Materials and Methods). As can be seen from Figure S5A in the SI, WT CymA is an electrically noisy channel because of the flexible N‐terminal loop that is weakly attached inside the channel from the periplasmic side.^[28]^ The putative movement of the N‐terminal loop into and back out of the pore creates rapid blockages of the channel. Removing the 15 amino acids long N‐terminal peptide silences the channel in an open form and this structural modification allows the experimental quantification of Ptm translocations.

**Figure 3:**
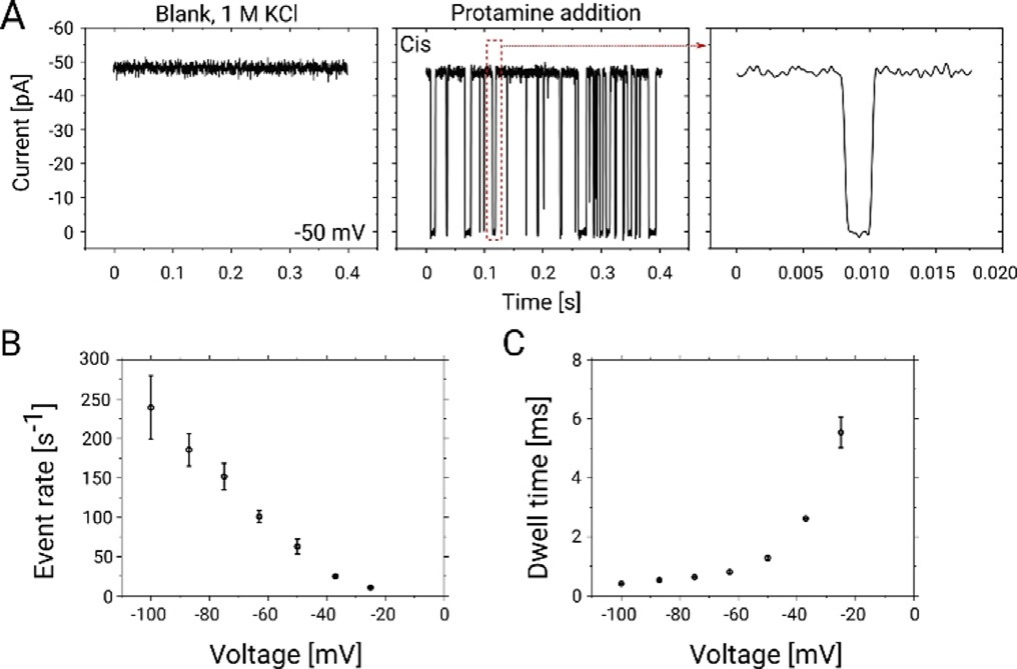
Single channel electrophysiology analysis. A) Ion current traces of a single reconstituted ΔCymA channel in a DPhPC bilayer for 1 m KCl at an applied potential of −50 mV revealing a silent channel (left panel). The protein was inserted from the *cis* side and the potential is applied from the *trans* side while the *cis* side is grounded. The middle panel shows a trace after addition of 1 μm Ptm on the *cis* side of the chamber while the rightmost panel magnifies a single Ptm translocation event. B, C) Statistical analysis of the ion‐current fluctuations in the presence of 1 μm Ptm as shown in (A). B) Event rates of Ptm. C) Dwell time on the *cis* side addition of protamine. All experiments were performed at 25 °C using 1 m KCl in 10 mm HEPES at pH 6 while the data were filtered at 2.5 KHz.

The ΔCymA channel was inserted from the *cis* side. After a successful single‐channel reconstitution was achieved, 1 μm of the substrate Ptm was added to the *cis* side of the chamber and a transmembrane potential *V*_m_ of negative polarity was applied on the *trans* side. This resulted in discrete ion‐current blockages with complete closures of the pore current and resolvable dwell times in the millisecond range (Figure 3 A). Such a behaviour is expected as the Ptm molecule is strongly cationic (+21 *e*) and with the application of negative potential, the molecules are pulled through the pore due to the electrophoretic force. When the substrate is administered on the opposite side (*trans* side), a positive potential creates similar ion current blockages (Figure S6A), suggesting that a permeation from the other side is also feasible, with similar efficiency. Increasing the magnitude of *V*_m_ further increased the number of blockages in both cases (Figure S7 in SI), meaning that the molecules are pulled at a faster rate across the channel by virtue of a stronger electrophoretic force.

To quantify the findings, we have analysed the event rate *f*_e_ which specifies the number of events per second and the dwell (or residence) time *τ*_d_ of Ptm molecules in the channel that is obtained from an exponential fit of the dwell time distribution. For both quantities at least 1000 events were analysed for each transmembrane voltage applied in the range from ±20 to ±100 mV. In Figure 3 B,C, the values of *f*_e_ and *τ*_d_ obtained from the experiments performed after *cis*‐side addition of Ptm and negative applied voltages are shown. Larger negative voltages increase the number of events, *f*_e_, and decrease the dwell times, *τ*_d_. A similar trend was observed for insertion of Ptm at the *trans* side and positive applied voltages (Figure S6B,C). This behaviour confirms that by increasing the magnitude of the applied voltages, more molecules are dragged through the channel. In previous studies, a similar trend was observed for the permeation of shorter poly‐arginine molecules through hetero‐oligomeric channels and through the general diffusion channel OmpF.^[29]^

In a second set of electrophysiological measurements, we characterised the concentration‐driven relative permeation by using the so‐called reversal potential measurement technique[^30^, ^31^] (see Section 2 in the SI for details on Material and Methods). Starting with two compartments having 0.1 mm potassium sulfate separated by a ΔCymA channel embedded in a DPhPC bilayer, a concentration gradient of 0.25 mm Ptm sulfate was introduced on the *cis* side. In principle, the large‐size Ptm molecules are expected to have a lower electrophoretic mobility compared to that of the sulfate counterions. Application of a 0.25 mm salt gradient caused an ion flux leading to a quasi‐equilibrium transmembrane voltage, the so‐called reversal potential, of about −20 mV (see Figure S9 in the SI). The reversal potential approach is relatively simple to measure but less straightforward to analyse and interpret for more complex ions such as highly charged polypeptides. For the further analysis, we assumed the effective charge of a Ptm molecule to be +21 *e* due to the 21 arginine residues. Moreover, 11 mobile sulfate ions, each having a charge of −2 *e,* are assumed to roughly counterbalance the respective charges. As detailed in the SI, these charge states lead to a permeability ratio for K^+^/Ptm^21+^/SO_4_^2−^ of 1:1:48. This ratio implies that Ptm molecules as well as sulfate ions may permeate across the ΔCymA channel and that for each Ptm molecule 48 counterions are permeating until the reversal potential has balanced this flux. It can be assumed that divalent SO_4_^2−^ ions binding at the inner pore surface will retain the natural cation selectivity of the pore, unlike Mg^2+^ ions which change the pore preference to anions.^[27]^ For a 1 μm concentration gradient of protamine sulfate across the membrane, the approximate flux can be determined to be three molecules per second for protamine and 60 molecules per second for sulfate, which is in close agreement with the rates estimated from the fluorescence assay and from the single‐channel electrophysiology experiments (see above).

### Atomistic Details of the Protamine Translocation Mechanism by MD Simulations

Lastly, we studied the translocation of a Ptm molecule through the ΔCymA channel by using molecular dynamics simulations (see Section 3 in the SI for details on the Material and Methods). Two states of Ptm, i.e., the helical and the disordered conformation (Figure 1 A), were considered for the simulations in order to elucidate which one will be the best suitable form for translocation. Moreover, the intention was to determine the feasibility of a Ptm molecule translocation through the pore.

Initially, we carried out applied‐field MD simulations by placing both Ptm conformations at the extracellular side in separate simulations. A positive external bias voltage of 1 V was applied and the simulations were conducted for 4 and 6 μs for the helical and disordered conformations, respectively. As shown in Figure S10 in the SI, the C‐terminus of both Ptm structures enters the channel first. This orientation is likely caused by the presence of more densely packed arginines at this end compared to the N‐terminus, which is electrostatically favoured by acidic residues on the channel wall. The C‐termini of both Ptm conformations reach the middle of the channel within 1 μs simulation time, but full translocations have not been achieved in the remaining simulation times. It is clear that the limited but already long simulation times are not sufficient to observe full translocation events. Notably, a deformation in the β‐barrel of the channel was observed towards the extracellular side when the helical Ptm conformation was moving further into the channel (see Figure S11 in the SI). This finding suggests that Ptm is too bulky in its helical state and it has to become fully or partially unfolded into the disordered form before or while permeating through the channel.

**Figure 4:**
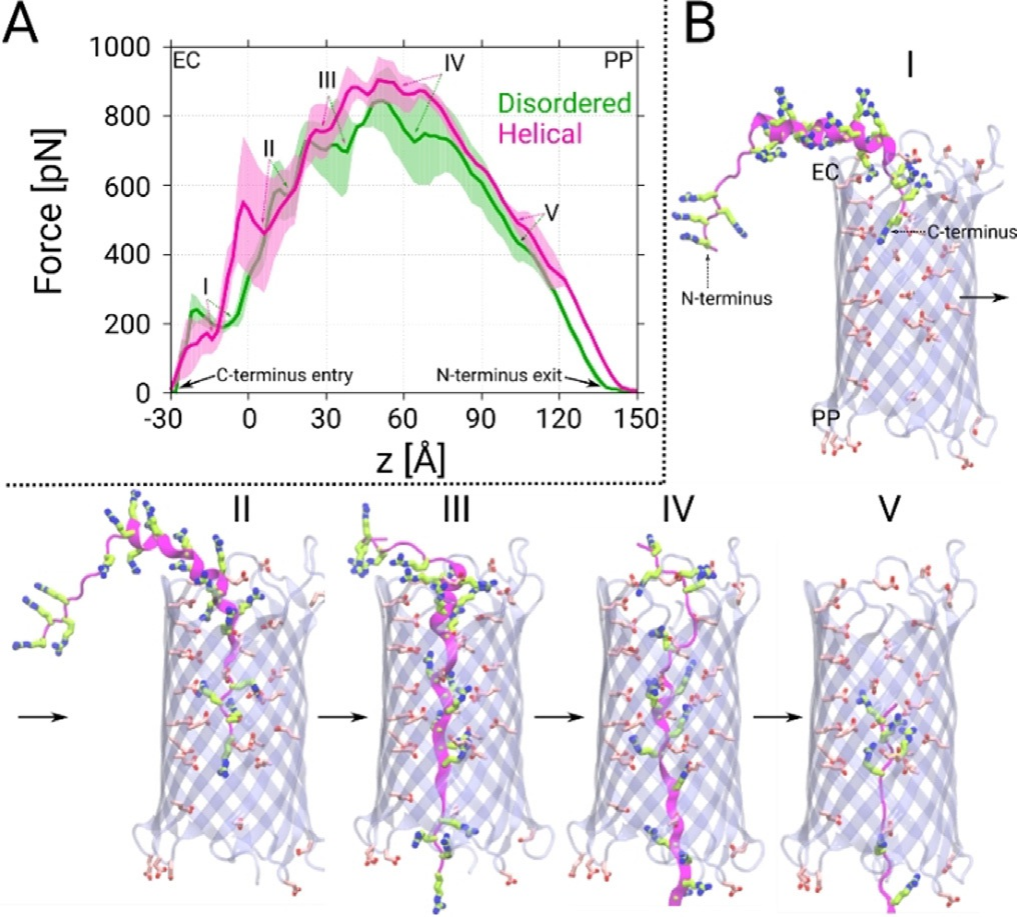
A) Force profiles as function of the reaction coordinate *z* based on constant‐velocity steered MD simulations of the disordered and helical forms of Ptm. Here, *z* represents the center‐of‐mass difference between the C_α_ atoms of the β‐barrel and the C‐terminus of Ptm along the *z*‐axis. The profiles shown are averages of three simulations and the error bars represent the standard deviations. The most probable metastable states found along the force profiles are denoted with Roman letters I to V in both cases. B) Representative snapshots depicting the helical Ptm conformations in the five identified metastable states. The acidic residues located along the channel axis are depicted as sticks (C: pink, O: red). The Ptm molecules are represented in the same manner as in Figure 1. Similar snapshots for the disordered Ptm are shown in Figure S12 of the SI.

To achieve insights into the complete permeation process, we additionally conducted steered MD simulations by pulling the C‐terminus of both Ptm conformations along the channel axis. The resulting force profiles shown in Figure 4 A suggest that the forces required to pull the helical form through the channel are slightly higher than those for the disordered form, i.e., the respective maximum forces approach 950 and 890 pN. Interestingly, the metastable states extracted from the permeation process reveal that helical Ptm unfolds into the disordered form before traversing the channel completely (Figure 4 B). This unfolding process is the reason behind the higher forces observed for the helical Ptm because the permeation of the disordered Ptm conformation occurs in a rather straightforward manner (see Figure S12 in the SI). This finding also supports our earlier claim that the helical Ptm conformation cannot directly traverse through the channel but has to transform into the less bulky disordered form. Moreover, the observed forces for the Ptm molecules are not extremely high in comparison to those observed for the “native” large‐molecule analytes of CymA, namely α‐ and β ‐cyclodextrins, since the highest values for the cyclodextrins are approximately 500 to 700 pN^[27]^ for similar steering velocities. Although the obtained simulated data are only average forces and not free‐energy profiles, the comparison of the absolute values certainly suggests that it is feasible for Ptm molecules to permeate through the ΔCymA channel. At the molecular level, the negatively charged acidic residues found along the interior surface of the channel facilitate the gliding of the arginine‐rich Ptm through the ΔCymA channel. Interestingly, Ptm molecules have four arginine‐rich regions (Figure 1 A) and entrance of each of these regions into the pore resulted in the four metastable states I to IV (Figure 4 B). It can be also suggested that states III and IV, during which most of the Ptm structure interacts strongly with the pore, must be the rate‐limiting states, which is supported by the highest necessary pulling forces.

## Discussion

The present study demonstrates that the combination of different complementary techniques allows an improved understanding of the translocation of large (5.1 kDa) polypeptides across the ΔCymA nanopore. The fluorescence study on liposomes unambiguously established that translocation of Ptm through the wild‐type and ΔCymA channels takes place with a translocation time in the order of seconds. Concentration‐driven fluxes can be estimated by using reversal potential measurements on planar lipid bilayers and revealed a permeability ratio of 48:1 for sulfate vs. Ptm molecules. Furthermore, single‐channel electrophysiology experiments revealed that the polycation can be pulled to and through the pore by electric fields and the comparison of these single‐molecule data with the ensemble measurements from the fluorescence assay also establishes that the observed events are actually due to translocation and not blockage. Higher negative voltages accelerate the translocation process and extrapolation of these results to zero voltage revealed rates of about one molecule s^−1^ for a gradient of 1 μm Ptm, in good agreement with the ensemble fluorescence kinetics. Moreover, the available high‐resolution structure allowed all‐atom modelling which revealed molecular details of the translocation process, most important, a preferential uptake of Ptm in its disordered conformation.

It is interesting to compare the present results to the translocation of neutral α‐cyclodextrin molecules (*M*_W_=973 Da) through ΔCymA. For the latter molecules, an extrapolation to similar concentration gradients reveals about 10 molecules per second with slightly shorter translocation times in the ms range. Note that the slightly larger β‐cyclodextrin (*M*_W_=1135 Da) is unable to permeate the membrane. Another comparison can be made with the antibiotic molecule kanamycin (*M*_W_=484 Da) for which very strong interactions with OmpF were observed during translocation.^[32]^ Although the molecular size is an order of magnitude smaller than that of Ptm and the pore has half the diameter of that of ΔCymA, the event rate is about the same whereas the dwell time is in the ms range. Overall, this trend suggests that in the case of bacterial channels, the molecular weight is only a rough exclusion criterion whereas the molecular shape and pore‐mediated interactions will finally control the translocation kinetics. Moreover, we conclude that even small concentration gradients are able to enforce the diffusion of large molecules like Ptm through narrow orifices.

## Conclusion

Our investigation revealed that a 5 kDa large peptide can permeate passively through mesoscopic channels. At concentration gradients relevant for antimicrobial activity (around 1 μm) about one molecule per channel and per second can enter bacteria, which is in the relevant range to cause activity inside the bacteria. Note that a common strategy in developing novel antibiotics is to create hybrid molecules. Crosslinking peptides, sugars, or nucleic acids as a “carrier” with the antibiotic as a cargo is expected to circumvent the resistance barrier. Our finding might encourage the development of hybrid antibiotics covalently attached to peptides. Some years ago, Lee and co‐workers pulled short peptides through a biological channel and correlated the resulting ion current flickering with the peptide sequence.^[33]^ By now this field is much more advanced and under favourable conditions even allows for a discrimination of single amino acids.^[34]^ Moreover, wide channels such as CymA allowing the passage of peptides are of key interest for peptide sequencing.

## Conflict of interest

The authors declare no conflict of interest.

## Supporting information

As a service to our authors and readers, this journal provides supporting information supplied by the authors. Such materials are peer reviewed and may be re‐organized for online delivery, but are not copy‐edited or typeset. Technical support issues arising from supporting information (other than missing files) should be addressed to the authors.

Supplementary
