# Supplementary material for: Large‐Peptide Permeation Through a Membrane Channel: Understanding Protamine Translocation Through CymA from Klebsiella Oxytoca
Source: Angew Chem Int Ed Engl. 2021 Mar 3;60(15):8089–94. doi: 10.1002/anie.202016943 (PMC8049027; doi:10.1002/anie.202016943)
Supplement: Supplementary file 1 — Supplementary [file ANIE-60-8089-s001.pdf]

Angewandte Chemie
Eine Zeitschrift der Gesellschaft Deutscher Chemiker

Supporting Information

Large-Peptide Permeation Through a Membrane Channel: Understanding Protamine Translocation Through CymA from *Klebsiella Oxytoca***

*Sushil Pangeni, Jigneshkumar Dahyabhai Prajapati, Jayesh Bafna, Mohamed Nilam, Werner M. Nau, Ulrich Kleinekathöfer, and Mathias Winterhalter**

anie_202016943_sm_miscellaneous_information.pdf

1 Membrane Tandem Assay

1.1 Materials and Methods

P-sulfonatocali(4)arene (CX4), lucigenin (LCG), Triton X-100, octyl-polyoxyethylene (O-POE), protamine sulfate and sodium phosphate were purchased from Sigma. 1-palmitoyl-2-oleoyl-sn-glycero-3-phosphocholine (POPC, 25 mg/mL dissolved im chloroform) was purchased from Avanti Polar Lipids. NAP-25 columns for purification were obtained from GE-Healthcare. Moreover, 2 mg/mL ΔCymA or WT-CymA in 1% octyl-polyoxyethylene (O-POE) was used from a previously purified batch of proteins.[1]

We followed the previously optimized assay for the reporter pair CX4/LCG.[2] Briefly, a lipid film was made in a 5 mL round bottom flask spreading about 100 µL of a stock solution (stock concentration: 25 mg/mL) of POPC at the surface during slow drying with nitrogen air and subsequently vacuum drying overnight. The film was hydrated with a final volume of 1 mL containing 10 mM NaH_2PO_4, 700 µM CX4, and 500 µM LCG and then stirred in a rota-evaporator at ambient temperature for about 20 minutes. The resulting liposome suspension was subjected to 20 freeze-thaw cycles (freezing in liquid nitrogen and thawing at 40 °C for 2 minutes each) to create unilamellar liposomes. CX4-LCG loaded liposomes were separated from free, non-encapsulated CX4 and LCG by using size-exclusion chromatography by using a NAP-25 column maintaining the same buffer condition.[2]

The size distributions of the obtained liposomes were determined by using a Zetasizer Nano (Malvern Instruments) and found to have a major peak at around 100 nm in diameter. This finding is in line with the preparation procedure for unilamellar liposomes, even if not all liposomes may be unilamellar.[3]

In a typical protamine uptake experiment, 20 µL liposome solution (stock solution 25 µM lipid) loaded with CX4/LCG (700/500 µM), in a 2-mL cuvette in 10 mM sodium phosphate solution, pH 7.0 at 25 °C, was prepared and the time-resolved fluorescence intensity was recorded. Subsequently, 45 nM ΔCymA (2.8 µL of 2 mg/ml of stock) was added to the solution to form ΔCymA-inserted liposomes (proteoliposomes). In a third step, protamine was added at a concentration specified by the respective experiment. At the end of the kinetic experiment, Triton X-100 (50 µL of neat detergent 1% Triton X-100) was added as a control to access the maximum fluorescence intensity after lysis of the liposomes. In all fluorimetric experiments, the fluorescence of the entrapped dye was monitored by excitation at 367 nm and emission at 500 nm on a Varian Eclipse spectrofluorimeter. For direct comparison, the fluorescence intensities were normalized according to $I = (I_t - I_0)/(lI_\infty - lI_0)$, where lI_0 is the initial intensity at t = 0, I_t is the intensity at time t, and lI_∞ is the final intensity, i.e., after the lysis of liposomes by Triton X-100.

In the above measurements, we used a 3.3 mM lipid solution to form unilamellar vesicles. Dynamic light scattering revealed a homogeneous liposome preparation of about 100 nm diameter as shown in Figure S1. Assuming about 0.5 nm^2 per lipid area will require about 10^7 lipid molecules to form a 100-nm bilayer vesicle.

Thus, 3.3 mM lipid corresponds to a vesicle concentration of about 30 nM. A concentration of 45 nM of porins will distribute theoretically about 1-1.5 porins per liposome; experimentally, there will be a distribution, with some liposomes containing no porins that will subsequently resist to be filled with Ptm. A concentration of 1 µM corresponds to 0.3 molecules inside a 100-nm vesicle and saturation will be quickly reached. The fluorophore and the cages are in excess concentration. To reach the equilibrium, passive diffusion will allow on average one Ptm molecule to enter and the kinetic is likely limited by entry through the porin.

1.2 Results

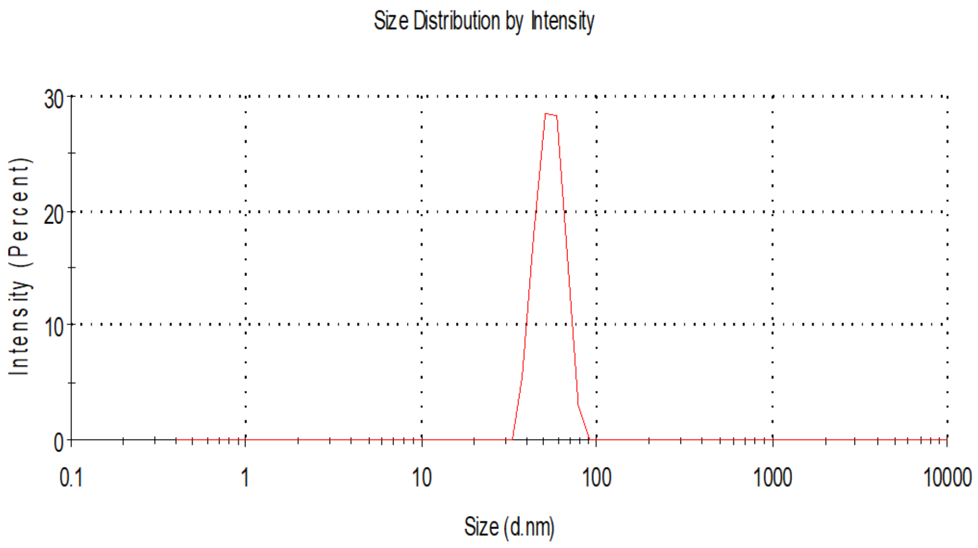

Figure S1: Size determination of the obtained liposomes (2 mM lipid) obtained by dynamic light scattering (DLS). The average size (diameter) of the liposomes was found to be 97 nm, with a uniform size distribution that is desirable for kinetic investigations by tandem membrane assay.

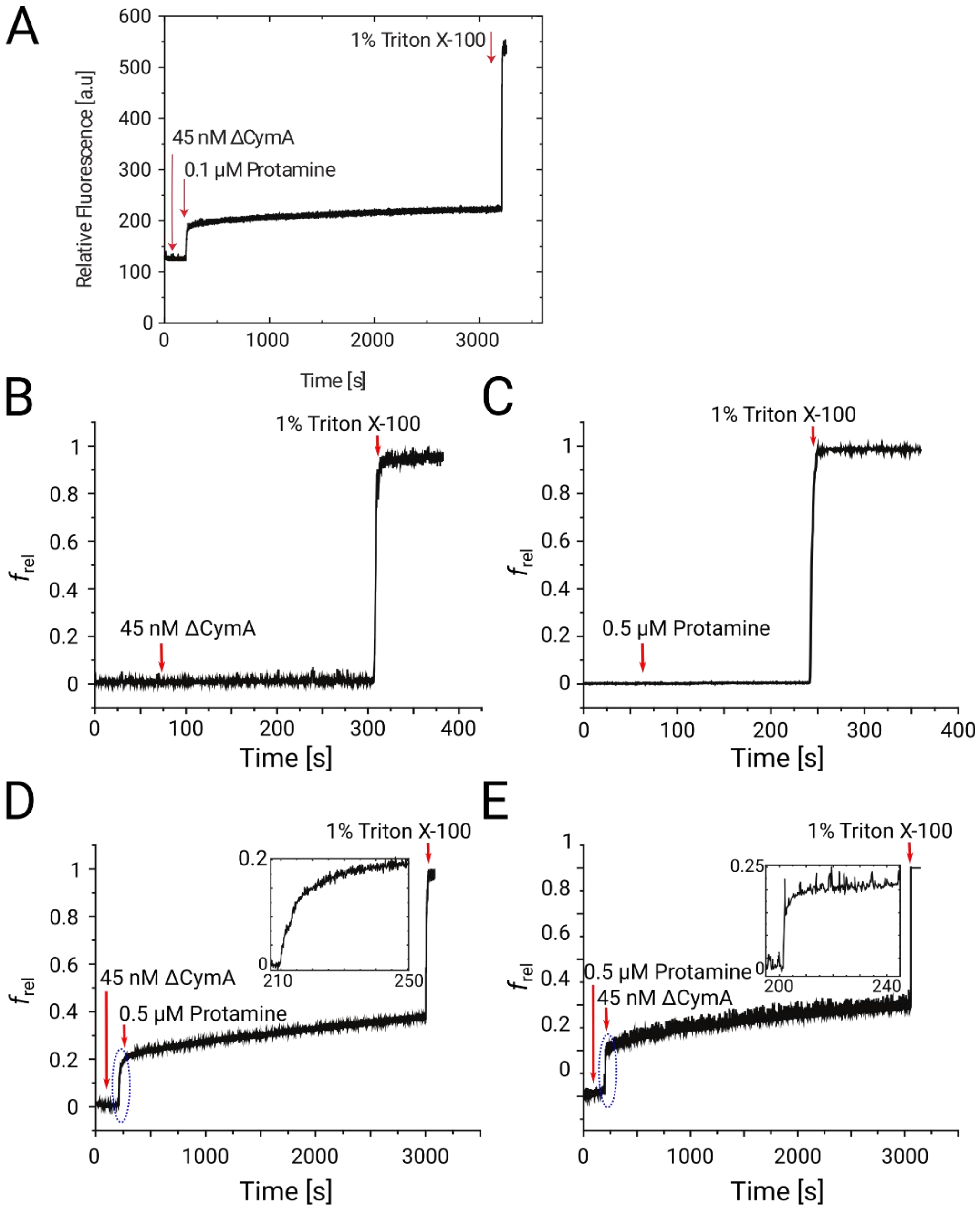

Figure S2: A) Raw data obtained from a typical fluorescence experiment. The fluorescence intensity (f_{rel}) shown in the figures are normalized using the value prior to Ptm addition (= 0) and the maximum fluorescence (= 1) after total release caused by detergent (Triton X-100) addition. B and C) Control measurements demonstrating the time courses of the normalized relative fluorescence intensity of CX4/LCG-loaded liposomes after addition of B) only 45 nM ΔCymA, C) only 0.5 µM Ptm. D and E) Time courses of relative fluorescence intensities (f_{rel}) of CX4/LCG-loaded liposomes after addition of D) first 45 nM ΔCymA followed by 0.5 µM Ptm and E) first 0.5 µM Ptm followed by 45 nM ΔCymA. The insets show the initial rise in f_{rel} observed after adding 0.5 µM Ptm and 45 nM ΔCymA in the respective experiments.

As a control, we first incorporated the channel protein ΔCymA into the liposomes containing the reporter pair CX4/LCG to form proteoliposomes. Measuring the fluorescence of the encapsulated liposome

complex afforded a flat baseline which excludes leakage of either CX4 or LCG through the pore (Figure S2B). Subsequent addition of Triton X-100 caused a complete vesicle lysis with a sudden release of the quenched fluorophore to a plateau. The difference between both extreme levels was normalized to 1 throughout the experiments. In a second control, we repeated the measurement with a new sample and added Ptm in the absence of any channel (Figure S2C). This did not cause any detectable increase in the fluorescence, which suggests that Ptm molecules do not self-permeate through the lipid membrane whereas the following addition of Triton X-100 resulted again in a fast increase due to lysis. In the actual translocation experiment, (Figure S2D), we first added ΔCymA pores followed by Ptm molecules leading to an initially rapid increase in the fluorescence followed by a slower rise. After 50 minutes, the vesicles were lysed with Triton X-100 to obtain the end point. In Figure S2E, we present the result of the experiment adding Ptm first and the ΔCymA channels thereafter; the change in the fluorescence was similar to that observed for the original sequence of addition. The selective fluorescence increases in the presence of both, Ptm molecules and ΔCymA channels, strongly suggests that Ptm permeation occurs selectively through the ΔCymA channels.

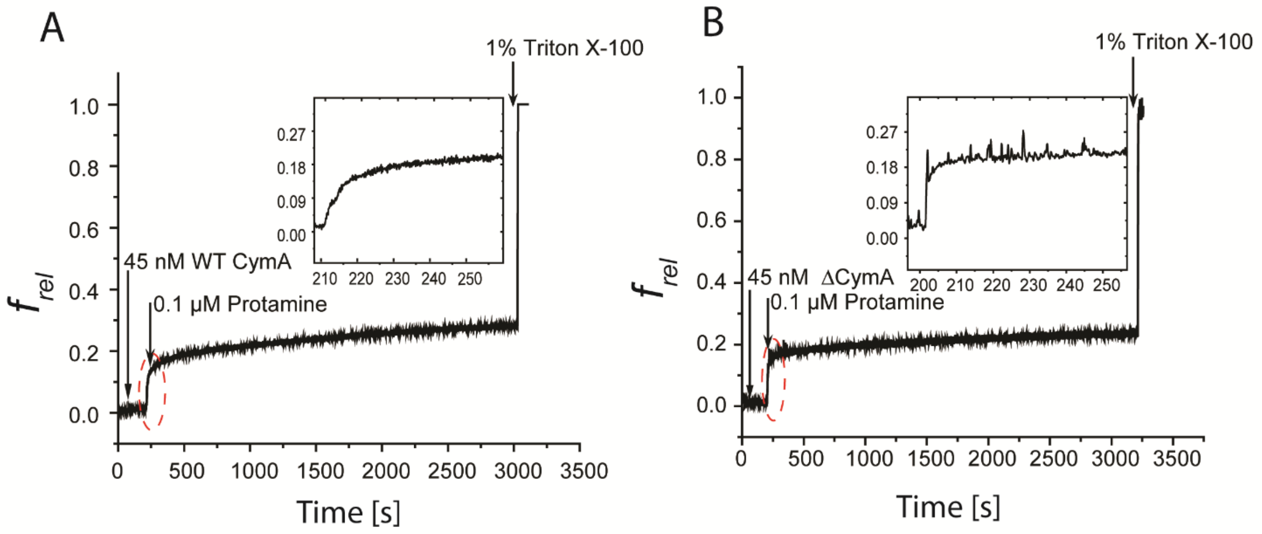

Figure S3: Difference in Ptm uptake for WT-CymA and ΔCymA channels in tandem membrane assays. Time course of the relative fluorescence intensity (f_{rel}) of CX4-LCG loaded liposomes after addition of 45 nM A) WT-CymA and B) ΔCymA followed by 0.1 µM Ptm. The insets show the initial rise in f_{rel} observed after adding 0.1 µM Ptm highlighting the slower initial rate in WT-CymA, where the N-terminal tail acts as a putative pore blocker.

2 Single Channel Electrophysiology

2.1 Materials and Methods: Planar Lipid Bilayer and Electrical Recording

Planar lipid bilayers conferring to Montal and Mueller were formed as published. [4–7] Briefly, an aperture in a Teflon septum with a diameter of 100-120 µm was pre-painted with hexadecane dissolved in n-hexane at 1-5% (v/v) and the cuvette compartments were dried for 30-35 min, in-order to eliminate the solvent. The bilayers were made with 1,2- diphytanoyl-sn-glycero-phosphatidyl-choline at a concentration of 5 mg/ml in n-pentane. We added a small amount (<µl) from a stock solution of the WT CymA or ΔCymA channel (1–2 mg protein/ml) to the cis (ground) or trans (amplifier) side chamber containing 2.5 ml salt solution. Standard Ag/AgCl electrodes were used to detect the ionic current. Moreover, the cis side electrode of the cell was grounded, whereas the trans side electrode was linked to the headstage of an Axopatch 700B amplifier, used for the conductance measurements in voltage clamp mode. The signals were filtered by an on-board low pass Bessel filter at 10 kHz and with a sampling frequency of 50 kHz. Examination of the current recordings was completed using Clampfit (Axon Instruments). The current-voltage relation of the individual experiments was determined from single averaged currents at given voltages. The concentration of the Ptm molecules used in the chamber was 1 µM made in 1 M KCl in 10 mM MES at pH 6 unless otherwise mentioned. The buffer used throughout the electrophysiology experiment was 1 M KCl in 10 mM MES at pH 6 unless otherwise stated.

2.2 Electrophysiology

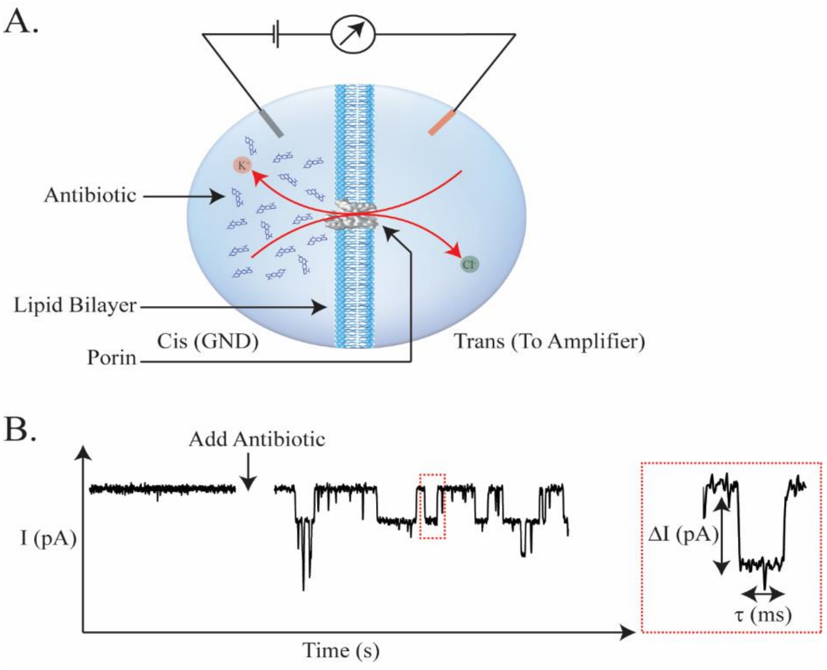

Figure S4: Schematic of electrophysiology experiment. A) Experimental setup, B) typical signal before and after the addition of substrate.

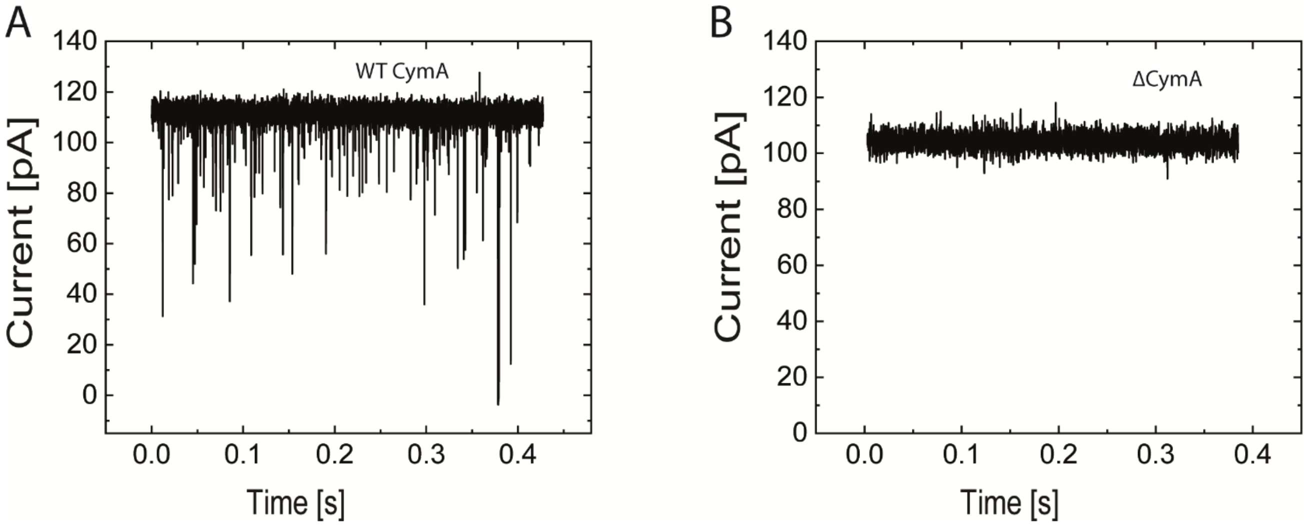

Figure S5: Ion Current traces of the blank A) WT CymA and B) ΔCymA channels in 1 M KCl and 10 mM MES at an applied membrane potential of 100 mV at pH 6.

WT CymA is an electrically noisy channel (Figure S5 A). As previously shown one can remove the 15 N-terminal residues hanging towards the inside of the loop, which are responsible for the noises (ion current blockages). Hence, quantification of small molecules translocation was tricky using WT CymA (Figure S5 A). Therefore, an electrically silent channel was created to be improve the transport properties of the channel.[1] When the 15

N-terminal residues were deleted (Figure 1 Main Text and Figure S5 B), the channel, now termed ΔCymA, is electrically silent.

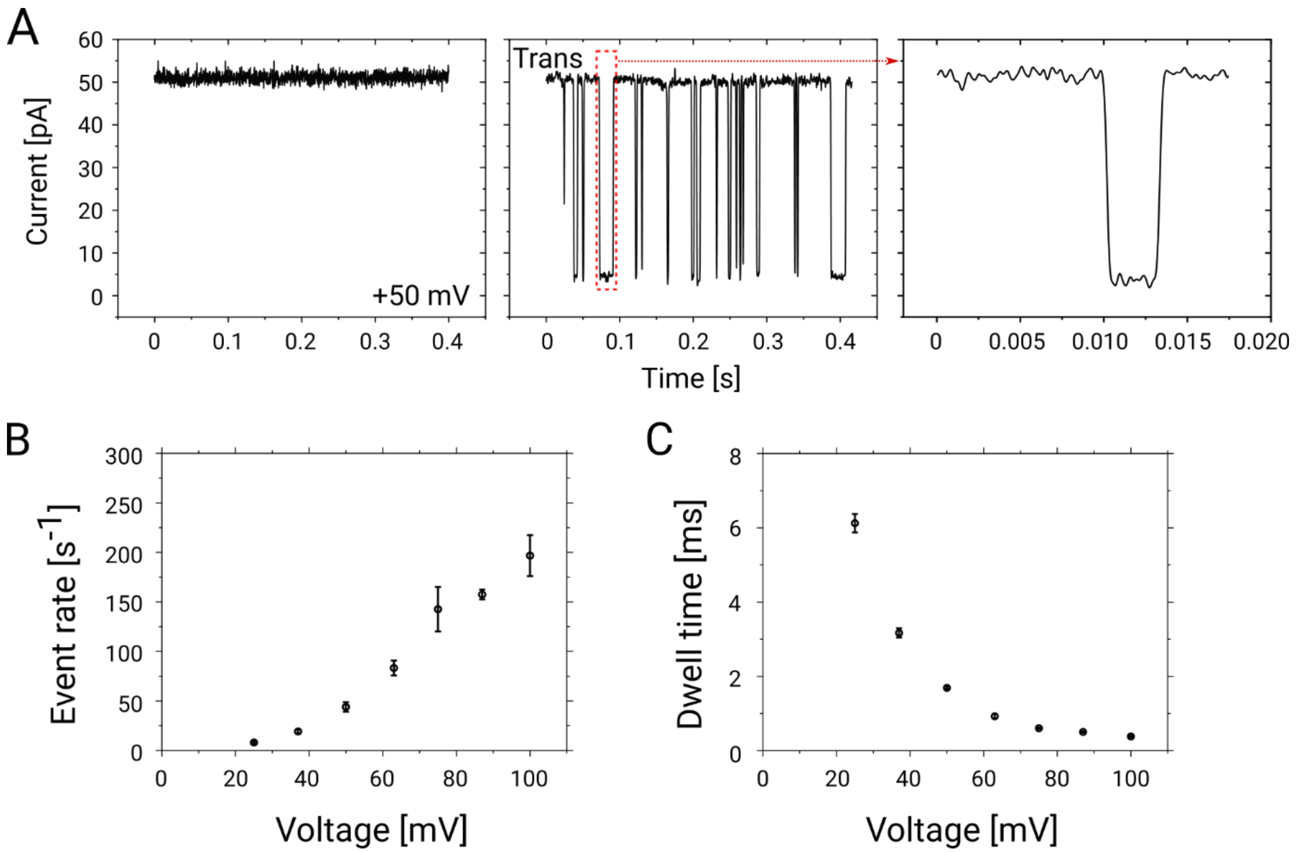

Figure S6. Single channel electrophysiology analysis. A) Ion current traces of a single reconstituted ΔCymA channel in a DPhPC bilayer for 1 M KCl at an applied potential of –50 mV revealing a silent channel (left panel). The protein was inserted from the cis side and the potential is applied from the trans side while the cis side is grounded. The middle panel shows a trace after addition of 1 µM Ptm on the trans side of the chamber while the rightmost panel magnifies a single Ptm translocation event. B and C) Statistical analysis of the ion-current fluctuations in the presence of 1 µM Ptm as shown in A. B) Event rates of Ptm C) Dwell time on the trans side addition of protamine. All experiments were performed at 25 ^{0}C using 1 M KCl in 10 mM HEPES at pH 6 while the data were filtered at 2.5 KHz.

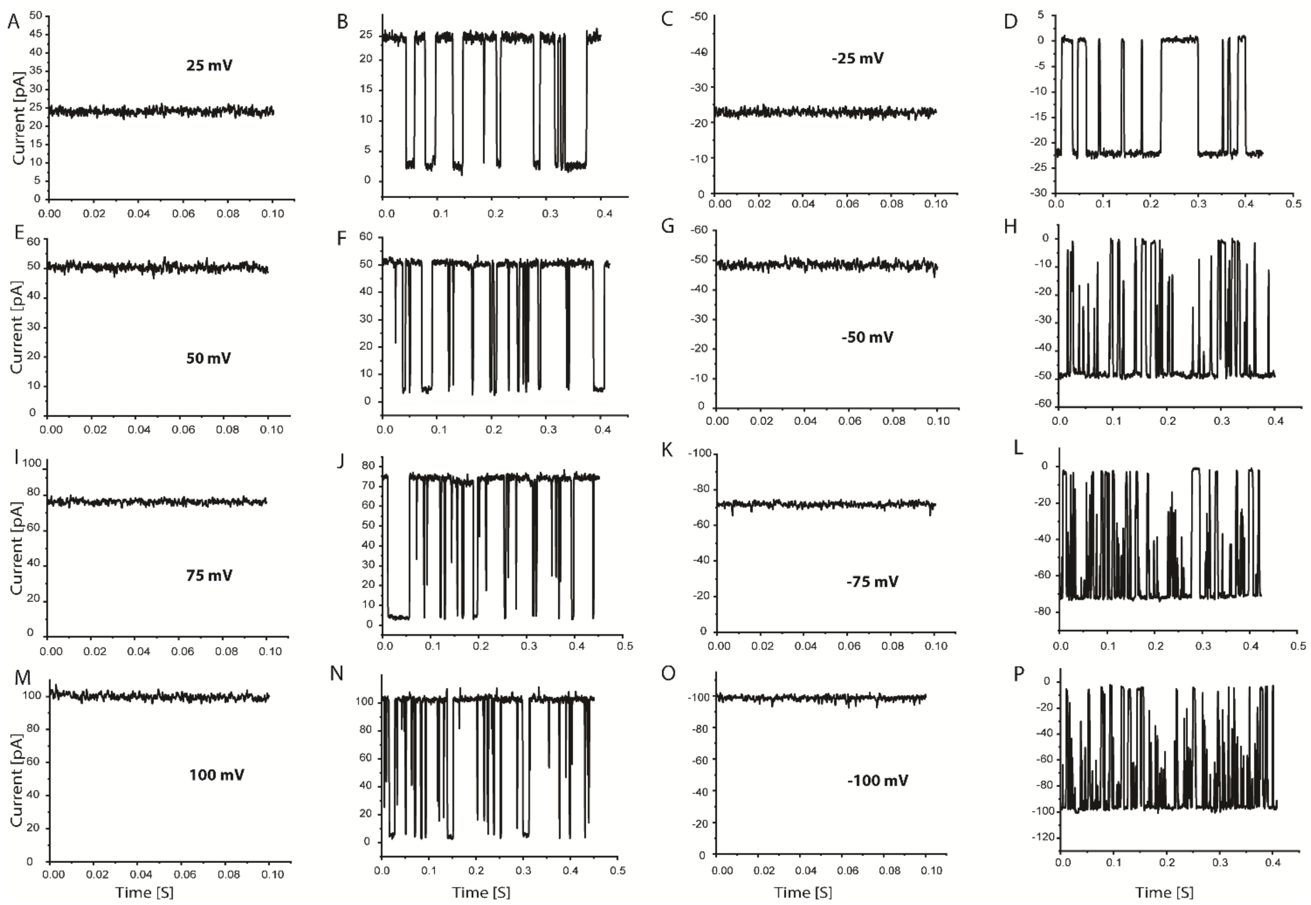

Figure S7: A & B) Ion current recordings of ΔCymA in the absence and the presence of the 1 µM Ptm (trans side addition) at voltage of +25 mV, respectively. C & D) Ion current recordings of ΔCymA in the absence and the presence of the 1 µM Ptm (cis side addition) at voltage of -25 mV, respectively. E-H) For the applied potential ± 50 mV. I-L) For the applied potential ± 75 mV. M-P) For the applied potential ± 100 mV. Experimental conditions are 1 M KCl, 10 mM MES and pH 6.

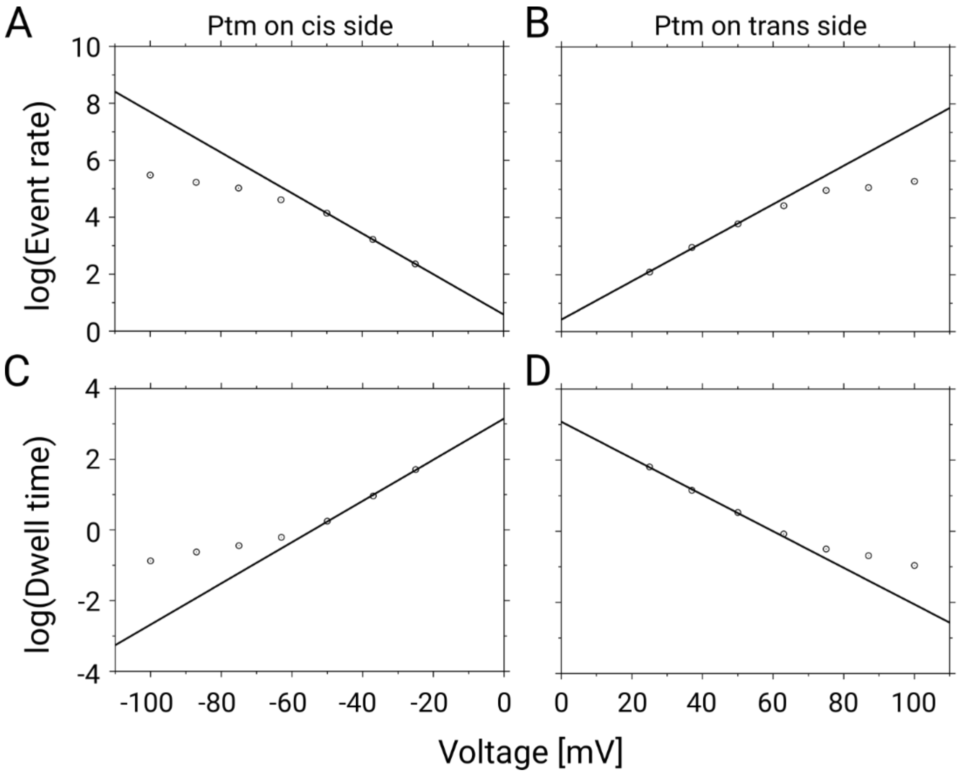

Figure S8: Event rates of Ptm at the A) cis and B) trans side of the ΔCymA channel with respect to the applied voltage are depicted on a semi-logarithmic scale. A linear regression for voltages lower than 50 mV of both polarities was conducted to extrapolate the event rate to 0 V. (C & D) Similarly, dwell times are shown on a semi-logarithmic scale and a linear regression was carried out to estimate the values at 0 V.

2.3 Reversal Potential Measurement

The reversal potential or Nernst potential is the membrane potential at which no net flow of a particular ion exists from one side of the membrane to other side in presence of a concentration gradient.[5,7]

To measure the reversal potential, we prepare a planar lipid bilayer setup as discussed earlier in the single channel electrophysiology measurement section. The porin of interest, i.e., ΔCymA, is inserted into the bilayer. Unlike in single channel measurements where one needs just a single channel, there is no issue with the number of channels in the membrane as the reversal potential does not depend on the number of channels inserted. We typically start with 0.1 mM potassium sulphate on both sides of the membrane to obtain an equilibrium and avoiding an accidental offset potential. Then, we record a blank IV curve by scanning voltages from ± 30 mV in steps of ± 10 mV and recording their respective currents. Next, we add 0.25 mM protamine sulphate on the cis side of the membrane. For this concentration gradient we again record an IV curve, and the reversal potential has been obtained by extrapolating the IV curve to zero membrane current.

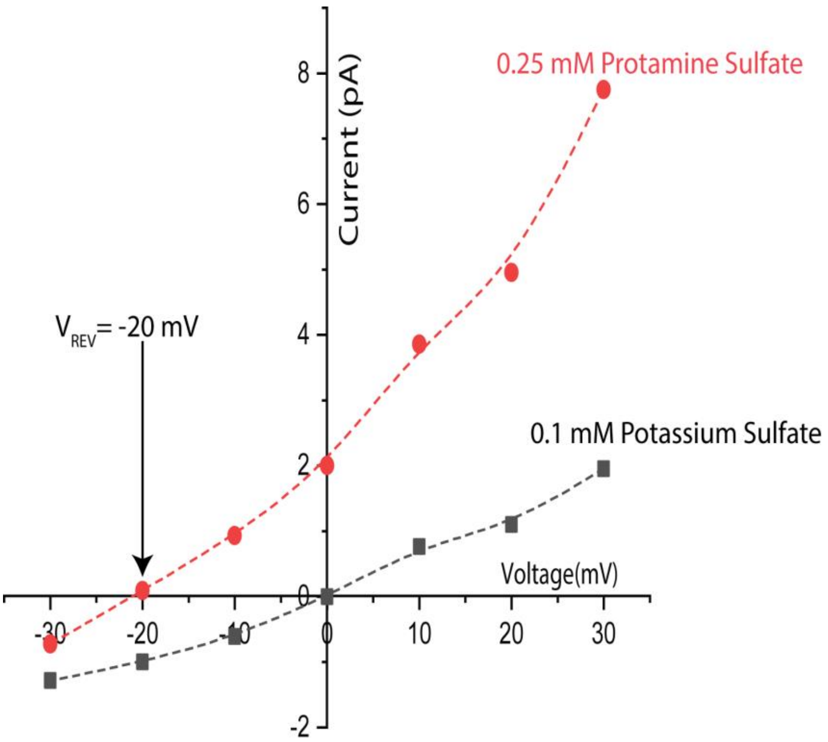

Figure S9: Trans-membrane permeability characterisation of protamine across ΔCymA.

To calculate the permeability ratios, we plug in the obtained reversal potential value in to the GHK current equation

$$\emptyset_x = \boldsymbol{P_x Z_x^2 \frac{V_m F^2}{RT} \frac{[c_x]_i - [c_x]_o e^{\left(\frac{-z_x V_m F}{RT}\right)}}{1 - e^{\left(\frac{-z_x V_m F}{RT}\right)}}} \quad (1)$$

where $\emptyset_x$ is the current density across the membrane carried by ion type x, measured in Am^{-2}, P_x is the membrane permeability of ion type x measured as ms^{-1}, z_x is the charge of species x, $V_m$is the membrane potential in volts, $[c_x]_i$ is the intracellular concentration of ion x and $[c_x]_o$ is the extracellular concentration of ion x, F denotes the Faraday constant 96485 C mol^{-1} , R the real gas constant 8.314 J mol^{-1} K^{-1} and T the temperature in Kelvin (in our case 298 K).

Table S1: Quantification of the protamine permeabilty through ΔCymA from Figure S8.

**Substrate**		**Cis side (mM)**				**Trans side (mM)**		**V_{rev} (mV)**	**Permeability ratios**
		K^+	SO_4^{2-}	Protamine	SO_4^{2-}	K^+	SO_4^{2-}		K^+ : SO_4^{2-} : $Protamine^{18+}$
**Protamine Sulphate**	**Blank**	0.1	0.1	0.0	0.0	0.1	0.1		
	**Gradient**	0.1	0.1	0.25	0.75	0.1	0.1	-20 ± 1.5	1 :48:1

3 Molecular Dynamics simulations

3.1 System Setup

First, the disordered and the helical structures of Ptm were built using ProBuilder and the I-TASSER webserver.[8] Then, two distinct system setups were built consisting of the ΔCymA channel immersed into a 1-palmitoyl, 2-oleoyl-sn-glycero-3-phosphocholine bilayer with a Ptm molecule placed towards the extracellular side in the two different conformations. The hydrogen atoms in all these molecules were treated as virtual sites to achieve an integration time step of 5 fs.[9–11] Then, both systems were solvated using TIP3P waters and 1 M KCl was added, leading to roughly 157,000 atoms in total. Subsequently, the systems were equilibrated for roughly 50 ns in several steps using the protocol defined in our previous study.[10] The temperature was maintained at 300 K and the pressure at 1 bar.

On both resulting systems, applied-field simulations[12] were performed in a first step with an external voltage of +1 V, which is calculated as the potential difference between the extracellular and the periplasmic sides of the channel. The simulations were performed for 4 and 6 µs for the helical and the disordered Ptm conformations, respectively. Subsequently, steered molecular dynamics (SMD) simulations[13] were carried out by pulling the C-terminus of the Ptm towards the periplasmic side of the channel in both systems. For this pulling, a reaction coordinate z was defines as the center of mass difference of the C_α atoms of the barrel and C-terminus of the Ptm molecule. The pulling was conducted at a constant velocity of 1 $Å \cdot ns^{-1}$ using a spring constant of 100 $kJ \cdot mol^{-1} \cdot nm^{-2}$. For both systems, simulations were repeated three time for a duration of about 200 ns. All simulations were performed using the GROMACS 2019.4 package[14] with the CHARMM36 force field.[15] The figures were generated using VMD.[16] The remaining simulation parameters were the same as in our previous study.[17]

3.2 Results

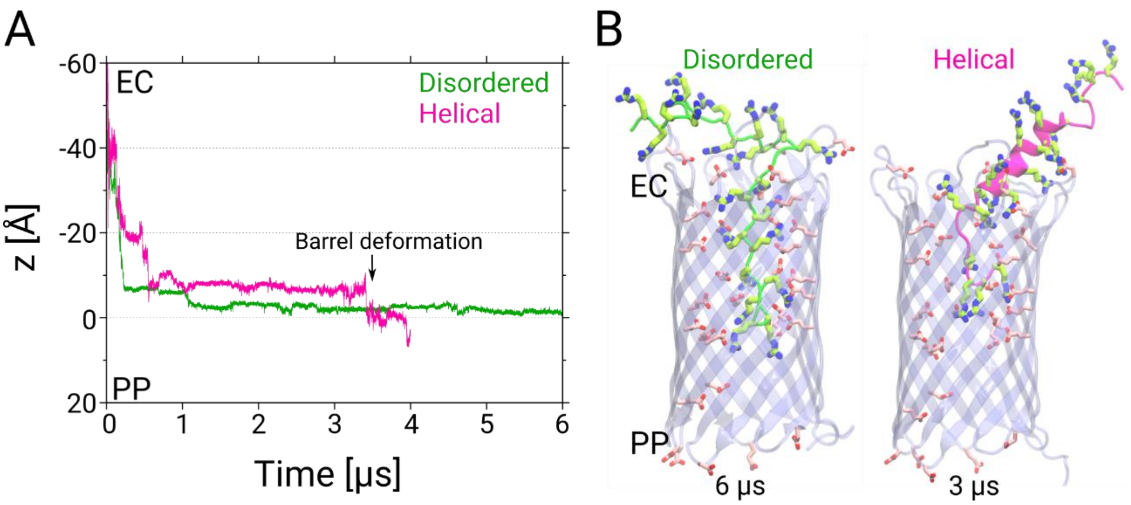

Figure S10: (A) The time evolution of the Ptm position with respect to the channel during the applied-field simulations of the disordered and helical structures. The Ptm position along the channel is represented using the reaction coordinate z which is calculated as center of masses difference between Cα atoms of the channel and C-terminus residue of the Ptm along the z-axis. (B) The snapshots representing the Ptm conformations after 6 and 3 μs for the disordered (left) and helical conformation (right), respectively. The acidic residues located along the channel are depicted as the sticks (C atoms: pink, O atoms: red). The Ptms molecules are represented in same manner as in Figure 1 of the main text.

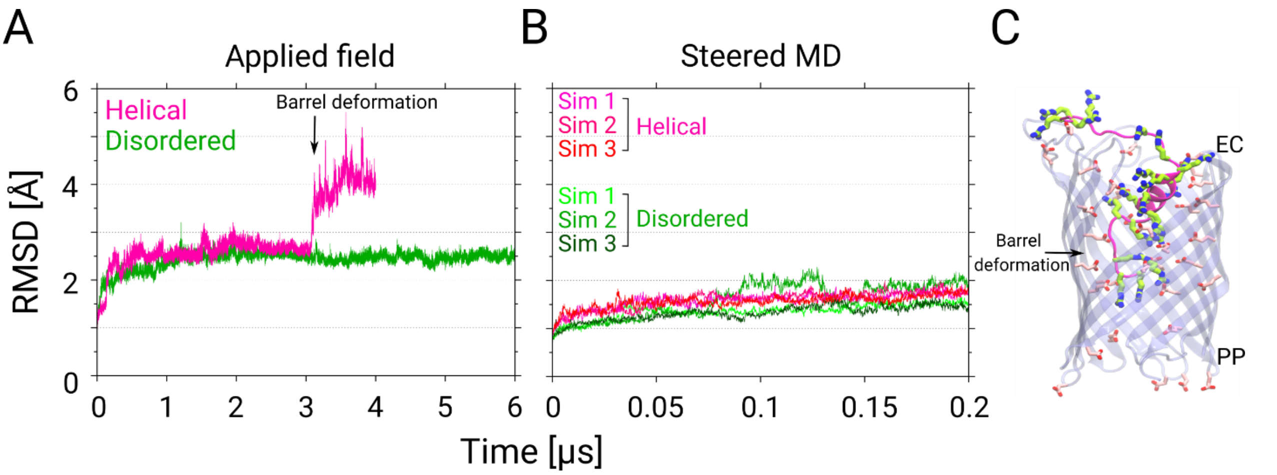

Figure S11: Root mean squared deviation (RMSD) of the ΔCymA channel calculated from (A) applied field simulations and (B) steered MD simulations. (C) The snapshot illustrates the deformation of the β-barrel towards the extracellular side of the ΔCymA channel after a 4 μs applied-field simulation of the helical Ptm.

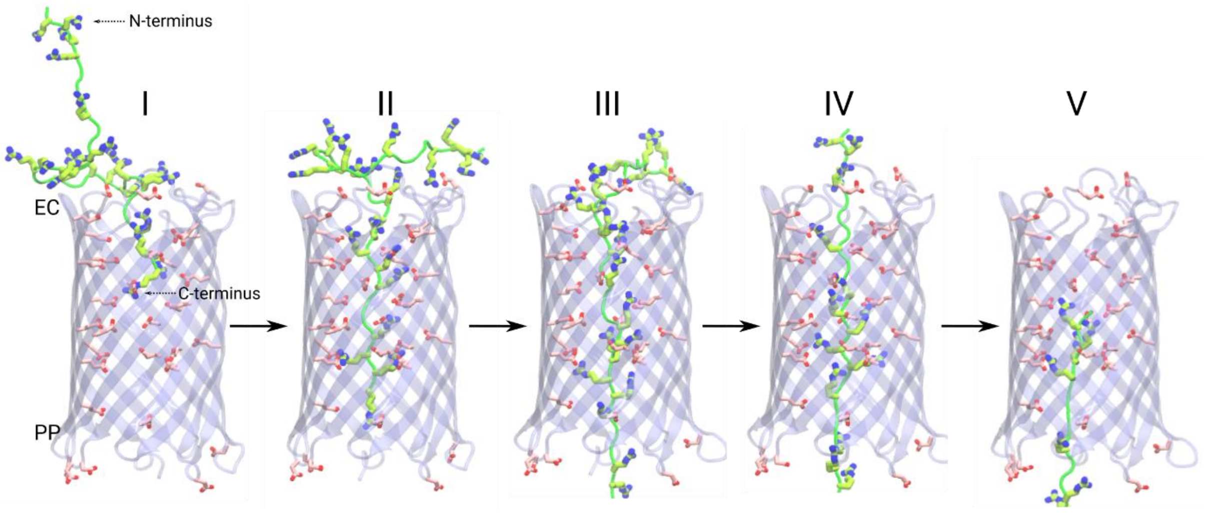

Figure S12: Representative snapshots depicting the disordered Ptm conformations in the five identified metastable states from the applied-field simulations.

References

[1] B. van den Berg, S. Prathyusha Bhamidimarri, J. Dahyabhai Prajapati, U. Kleinekathöfer, M. Winterhalter, *Proc. Natl. Acad. Sci.* **2015**, *112*, E2991–E2999.

[2] G. Ghale, A. G. Lanctôt, H. T. Kreissl, M. H. Jacob, H. Weingart, M. Winterhalter, W. M. Nau, *Angew. Chemie Int. Ed.* **2014**, *53*, 2762–2765.

[3] J. P. Colletier, B. Chaize, M. Winterhalter, D. Fournier, *BMC Biotechnol.* **2002**, *2*, DOI 10.1186/1472-6750-2-9.

[4] M. Montal, P. Mueller, *Proc. Natl. Acad. Sci.* **1972**, *69*, 3561–3566.

[5] I. Ghai, A. Pira, M.A. Scorciapino, I. Bodrenko, L. Benier, M. Ceccarelli, M. Winterhalter, R. Wagner, *J. Phys. Chem. Lett.* **2017,** 8, 1295-1301

[6] T. Gutsmann, T. Heimburg, U. Keyser, K.R. Mahendran, M. Winterhalter, *Nature Protocols* **2015,** 10, 188-198

[7] J. A. Bafna, S. Pangeni, M. Winterhalter, M. A. Aksoyoglu, *Biophys. J.* **2020**, *118*, 2844–2852.

[8] Y. Zhang, *BMC Bioinformatics* **2008**, *9*, 40.

[9] K. A. Feenstra, B. Hess, H. J. C. Berendsen, *J. Comput. Chem.* **1999**, *20*, 786–798.

[10] J. D. Prajapati, C. J. Fernández Solano, M. Winterhalter, U. Kleinekathöfer, *J. Chem. Theory Comput.* **2017**, *13*, 4553–4566.

[11] J. D. Prajapati, C. J. F. Solano, M. Winterhalter, U. Kleinekathöfer, *J. Phys. Chem. B* **2018**, *122*, 1417–1426.

[12] A. Aksimentiev, K. Schulten, *Biophys. J.* **2005**, *88*, 3745–3761.

[13] S. Izrailev, S. Stepaniants, M. Balsera, Y. Oono, K. Schulten, *Biophys. J.* **1997**, *72*, 1568–1581.

[14] M. J. Abraham, T. Murtola, R. Schulz, S. Páll, J. C. Smith, B. Hess, E. Lindahl, *SoftwareX* **2015**, *1–2*, 19–

25.

[15] L. Huang, B. Roux, *J. Chem. Theory Comput.* **2013**, *9*, 3543–3556.

[16] W. Humphrey, A. Dalke, K. Schulten, *J. Mol. Graph.* **1996**, *14*, 33–38.

[17] V. K. Golla, J. D. Prajapati, M. Joshi, U. Kleinekathöfer, *J. Chem. Theory Comput.* **2020**, *16*, 2751–2765.
